# Rosiglitazone Use and the Risk of Bladder Cancer in Patients With Type 2 Diabetes

**DOI:** 10.1097/MD.0000000000002786

**Published:** 2016-02-12

**Authors:** Eugene Han, Suk-Yong Jang, Gyuri Kim, Yong-ho Lee, Eun Yeong Choe, Chung Mo Nam, Eun Seok Kang

**Affiliations:** From the Department of Internal Medicine, Division of Endocrinology and Metabolism (EH, GK, Y-HL, ESK); Graduate school (EH, GK, EYC, ESK); Institute of Endocrine Research (Y-HL, ESK); and Department of Preventive Medicine, Yonsei University College of Medicine, Seoul (S-YJ, CMN); and Endocrinology and Metabolism Clinic, Internal Medicine, International St. Mary's Hospital, Incheon, South Korea (EYC).

## Abstract

Patients with diabetes have a higher incidence of bladder cancer; however, the association between thiazolidinedione use and bladder cancer risk has been controversial. We aimed to investigate whether pioglitazone or rosiglitazone use is associated with bladder cancer risk in patients with type 2 diabetes mellitus.

This nationwide nested case-control study used data set obtained from the Korean National Health Insurance Service National Sample Cohort 2002 to 2013. Among the 47,738 patients with incident diabetes, 85 cases of newly diagnosed bladder cancer and 850 controls (1:10 matched by age, sex, index year, and diabetes diagnosis year) were recruited. Type 2 diabetes mellitus and bladder cancer were diagnosed using the International Statistical Classification of Diseases and Related Health Problems, 10th Revision code.

More cases of bladder cancer were diagnosed in men (81.2%), and the stratified age peaked at 70 to 79 years old. Exclusive rosiglitazone use raised the incidence of bladder cancer (odds ratio [OR] = 3.07, 95% confidence interval [CI ] = 1.48–6.37). The risk of bladder cancer started to increase after less than 3 months use (OR = 3.30, 95% CI = 1.02–10.70) and peaked at 3 to 12 months of rosiglitazone use (OR = 4.48, 95% CI = 1.51–13.31). Patients were first exposed to exclusive rosiglitazone within 1 year (OR = 11.74, 95% CI = 2.46–56.12) and those who had consistently used it for 1 year (OR = 4.48 95% CI = 1.51–13.31), had higher risks of bladder cancer compared with nonthiazolidinedione users. Neither pioglitazone use nor exclusive pioglitazone use were associated with an increased incidence of bladder cancer.

Rosiglitazone use is associated with an increased risk of incident bladder cancer independent of age and sex in patients with type 2 diabetes mellitus. The highest odds of bladder cancer in rosiglitazone users was seen in those with <1 year of exposure.

## Abstract 2

Supplemental Digital Content is available in the text

## INTRODUCTION

The prevalence of type 2 diabetes mellitus (T2DM) has increased dramatically worldwide.^1^ Metformin is recommended as a first-line pharmacologic therapy for T2DM management,^2^ however, T2DM is a progressive disease that often requires a second-line agent, such as thiazolidinedione (TZD).^2^ Thiazolidinediones are ligands of peroxisome proliferator-activated receptor (PPAR) gamma, a member of the nuclear receptor superfamily of transcription factors.^3^ As PPAR gamma plays essential roles in improving glucose tolerance and insulin sensitivity, TZDs are referred as “insulin sensitizers.”^4,5^

Bladder cancer has a higher incidence in subjects with diabetes,^6^ but the association between TZD use and bladder cancer risk remains controversial.^7–12^ The concern of bladder cancer risk with TZD was first noticed in the Prospective Pioglitazone Clinical Trials in Macrovascular Events (PROactive), with 14 cases of bladder cancer in the pioglitazone group compared with 5 in the placebo group (relative risk 2.83; 95% confidence interval [CI] 1.02–7.85, *P* = 0.040).^11,12^ In addition, large, observational epidemiological studies conducted in Europe, concluded that pioglitazone was associated with an increased risk of bladder cancer.^7,8^ However, others reported no association between the risk of bladder cancer and TZD use.^9,10^ A 10-year epidemiological study conducted by the University of Pennsylvania and Division of Research at Kaiser Permanente Northern California was undertaken at the request of the US Food and Drug Administration as a safety study, and the authors finally announced that there was no association between bladder cancer risk and the duration of pioglitazone use, although an interim report described a slightly increased risk in patients prescribed pioglitazone for more than 24 months.^13,14^

Most studies have analyzed data derived from Caucasian subjects and mainly focused on pioglitazone.^6–13,15^ It is necessary to evaluate the association between TZDs, including pioglitazone and rosiglitazone and bladder cancer in Asian populations. In the present study, we analyzed data from the Korean National Health Insurance Service (NHI), which is a nationwide cohort study with a nationally representative sample of the Korean population conducted by the Korea Centre for Disease Control and Prevention to regularly assess the health status of general civilians. Our aim was to investigate the association between TZD use and the incidence of bladder cancer in a nested case-control study of the T2DM population.

## METHODS

### Data Source

We used 2002 to 2013 data from the South Korean National Health Insurance Service National Sample Cohort (NHIS-NSC). This is nationwide, representative 2%, stratified, random sample of a Korean population with a baseline of 1,025,340 subjects in 2002 to a final value of 1,014,730 subjects in 2013. It contains all inpatient and outpatient medical claims data, including personal information, prescription drugs, diagnostic and treatment codes, and primary and secondary diagnosis codes. Mortality data were provided by the National Statistical Office in Korea.^16^ Under universal medical coverage, all medical claims data are collected by the NHI as a monopolistic health insurer in Korea. Therefore, all subjects in the NHIS-NSC were maintained until 2013 except for follow-up loss due to death or disqualification from the NHI (eg, emigration). The ethics committee of the Yonsei University College of Medicine approved this study (4–2015–0579).

### Incident Diabetes Cohort

Within the NHIS-NSC, among subjects without any prescribed antidiabetic agent in 2002, we selected patients who were first prescribed oral antidiabetic agents from 2003 to 2013 so that all study subjects had at least 1 year, in which they were antidiabetic agent free.^7^ We further excluded patients whose first antidiabetic agent was insulin and patients who were <40 years old at the time of first antidiabetic prescription. Finally, patients with type 1 diabetes mellitus or pre-existing T2DM patients were excluded. The date of incident diabetic cohort entry was defined as the date of first antidiabetic agent prescription. The last follow-up date was defined as the date of bladder cancer diagnosis, death, disqualification from NHI, or December 31, 2013, whichever occurred. The final incident diabetic cohort consisted of 47,738 patients.

### Case-Control Patient Selection From the Incident Diabetic Cohort

In this nested case-control study with risk-set sampling (or incidence-density sampling), control patients were chosen from those in the incident diabetic cohort who were at risk of becoming a case at the time they were diagnosed.^17^ The index date was defined as the date 1 year before the date of bladder cancer diagnosis. Case patients were identified using the International Statistical Classification of Diseases and Related Health Problems, 10th Revision (ICD-10).^18^

Case inclusion criteria were first-time diagnosed bladder cancer (ICD C67.0-C67.9); at least 1 year of a latent period after T2DM diagnosis; without prior diagnosis of urinary tract cancer (kidney, renal pelvis, ureter, or bladder cancer, ICD C64.-C67.) within 5 years before the date of bladder cancer diagnosis; and presence of clinical codes for treatment (open surgery, transurethral bladder surgery, chemotherapy, radiation, chemotherapeutic instillation of bladder), codes for death due to bladder cancer, codes for diagnosis (biopsy, bladder tumor antigen test, cystoscopy), or codes for death within 6 months of bladder cancer diagnosis. Exclusion criteria were a prior diagnosis of urinary tract cancer within 5 years before bladder cancer diagnosis. Control patients were randomly selected from the case risk set at a 1:10 ratio after they were matched on a 5-year interval age group at both 2002 and the time of T2DM diagnosis, sex, and date of T2DM diagnosis within 1 year. For random selection from the matched risk set, we applied the same exclusion criteria as that of the case group.

### Thiazolidinedione Exposure

For all analysis, prescription information before the index date was used for the identification of drug use. Rosiglitazone use was defined as at least one prescription between cohort entry and the index date. The same definition of drug use was applied to all types of drugs including nonantidiabetic drugs. Due to the absence of case patients who had used both rosiglitazone and pioglitazone, study patients were classified into 3 categories of TZD use never used TZDs, exclusive rosiglitazone use, and nonexclusive rosiglitazone use including exclusive pioglitazone users.

As a secondary analysis, cumulative exposure and time of exposure among exclusive rosiglitazone users were examined. The cumulative duration was defined as the number of days between the first and last prescription plus the duration of last prescription up to the index date.^7^ Due to the absence of case patients who had used rosiglitazone >24 months, cumulative duration >12 months was the highest level in its category. The cumulative rosiglitazone dose was computed by summing the values of all prescriptions obtained by multiplying the daily dosage by prescribed days for each prescription. Then, the cumulative doses of rosiglitazone were categorized into tertiles. To consider the time of exposure, the time of first exposure to rosiglitazone was defined as the distance between the date of the first rosiglitazone prescription and the index date. We separated the above analyses into 2 distinct TZD uses: rosiglitazone and pioglitazone.

### Statistical Analysis

Characteristics of case and control patients are presented as numbers with percentages for categorical variables and as means with standard deviations for continuous variables. Conditional logistic regression was conducted to estimate odds ratios (ORs) and 95% CIs to assess the association between rosiglitazone use and the risk of bladder cancer. In addition to matching variables, we adjusted for urolithiasis and other urinary tract disease (ICD codes N20.-N23., N3.), renal disease (ICD codes N03.2-N03.7, N05.2-N05.7, N18., N19., I12.0, I13.1), alcoholic liver disease and alcohol related metal and behavioral disorders (ICD codes K70., F10.), chronic lower respiratory disease except for asthma (ICD codes J40.-J47., J47.), congestive heart failure (ICD codes I43., I50., I09.9, I11.0, I13.0, I13.2, I25.5, I42.0, I42.5-I42.9), Charlson comorbidity score calculated excluding diabetes and the comorbid diseases listed above, other antidiabetic drugs use (metformin, sulfonylurea, dipeptidyl peptidase [DPP]-IV inhibitor, and insulin), aspirin use, statin use, household income as a continuous variable (from 0 for medical aid to 10 for the highest income level), and residential area.^19^ No information on smoking habits was collected for the NHIS-NSC; therefore, we assumed that chronic lower respiratory disease indirectly represented cigarette smoking. A sensitivity analysis was conducted that excluded patients with a diagnostic code alone, those who died within 6 months of bladder cancer diagnosis, and those with any previous cancer history. All covariates were assessed by all information prior to the index date. All statistical analyses were conducted using SAS (Version 9.4, SAS Institute Inc, Cary, NC). A *P* value <0.05 was considered statistically significant.

## RESULTS

**FIGURE 1:**
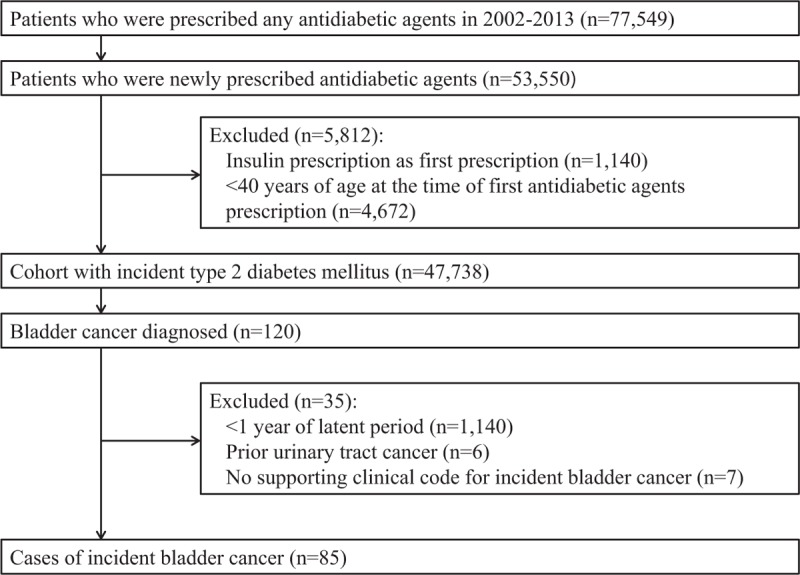
Flow of subjects through study.

From the original cohort of 47,738 subjects with incident T2DM, 120 patients with a first-time diagnostic code of bladder cancer were selected. We further excluded 35 patients who did not satisfy inclusion criteria. There were 85 final case patients, and the median follow-up time was 2880 days (Figure 1).

### Characteristics of Bladder Cancer Cases and Matched Controls

Table 1 shows the baseline characteristics of the 85 bladder cancer case patients and 850 control patients. Matching variables, including age, sex, and year at T2DM diagnosis were evenly distributed between case and control patients. Participants were predominantly men (81.2%) and more than half (56.5%) were over 60 years old. Compared with the control group, bladder cancer case group patients had more urolithiasis and previous cancers excluding urinary tract cancer. Chronic lower respiratory disease and Charlson comorbidity score were slightly increased in case group.

**TABLE 1:**
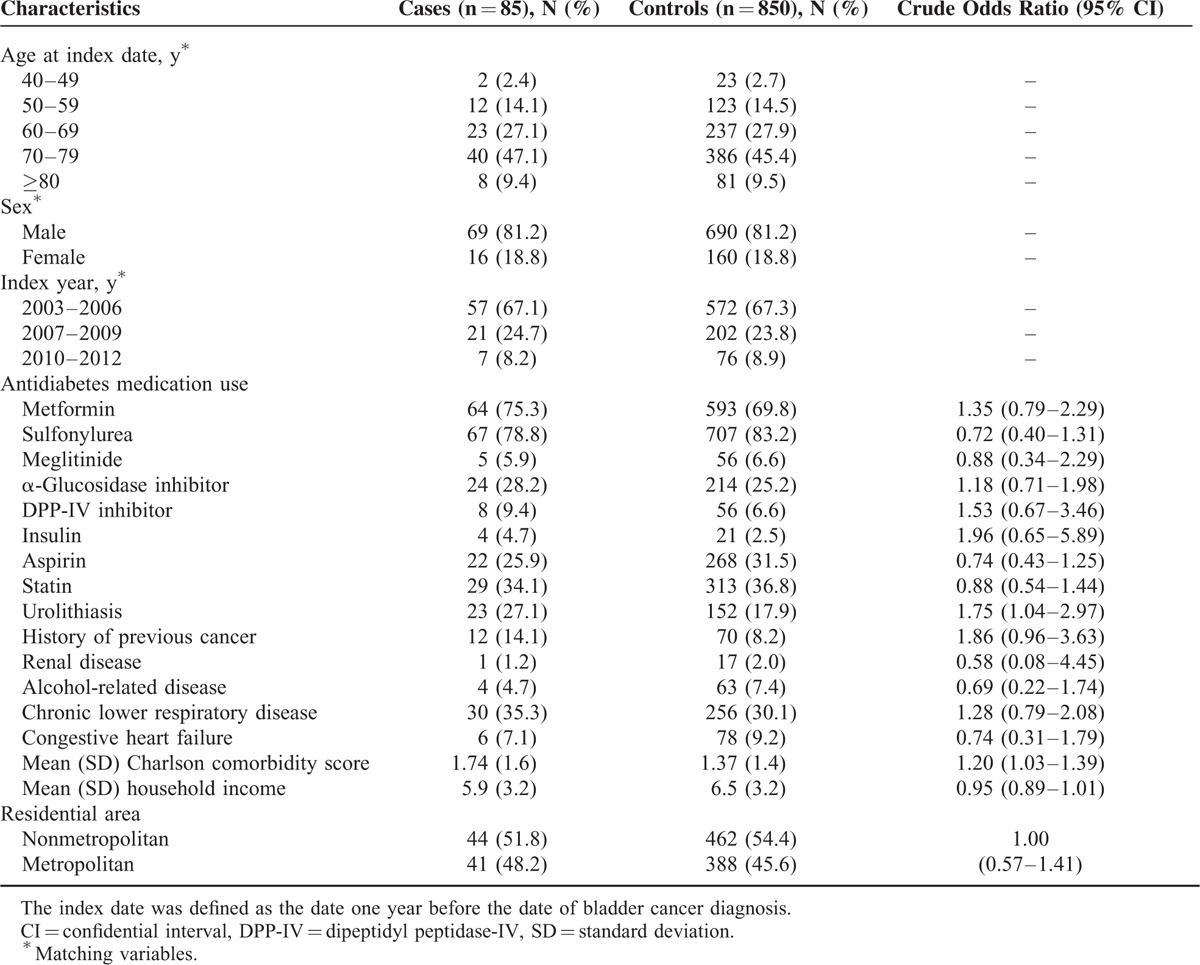
Baseline Characteristics of Bladder Cancer Cases and Matched Controls

### Relationship Between Thiazolidinedione Use and Bladder Cancer

Exclusive rosiglitazone use raised the incidence of bladder cancer by approximately threefold compared to no use of any TZD (adjusted OR [AOR] 3.07; 95% CI 1.48–6.37, Table 2). To evaluate the dose-response relationship between exclusive rosiglitazone use and the rate of bladder cancer, we stratified cumulative duration and rosiglitazone dose. The risk of bladder cancer increased after less than 3 months use (AOR 3.30; 95% CI 1.02–10.70) and peaked at 3 months to 12 months of exclusive rosiglitazone use (AOR 4.48; 95% CI 1.51–13.31). For those who used the drug more than 12 months, the risk was relatively lower compared to shorter time period. Similar increased risks were observed in lowest (AOR 3.67; 95% CI 1.24–10.88) and middle tertiles (AOR 4.03; 95% CI 1.25–12.96) of cumulative rosiglitazone dose. The cumulative dose of rosiglitazone 4 mg tablet once daily for 3 months is 360 mg, and the same dose for 9 months is 1080 mg. Therefore, the tertiles of cumulative dose corresponded to those of cumulative duration. As for TZD use timing, patients with their first exposure to exclusive rosiglitazone in 13 to 24 months (AOR 11.74; 95% CI 2.46–56.12) and patients with recent use within 1 year of the index date (AOR 3.98; 95% CI 1.18–13.41) had higher risk of bladder cancer. However, neither ever pioglitazone use nor exclusive pioglitazone use was associated with an increased incidence of bladder cancer.

**TABLE 2:**
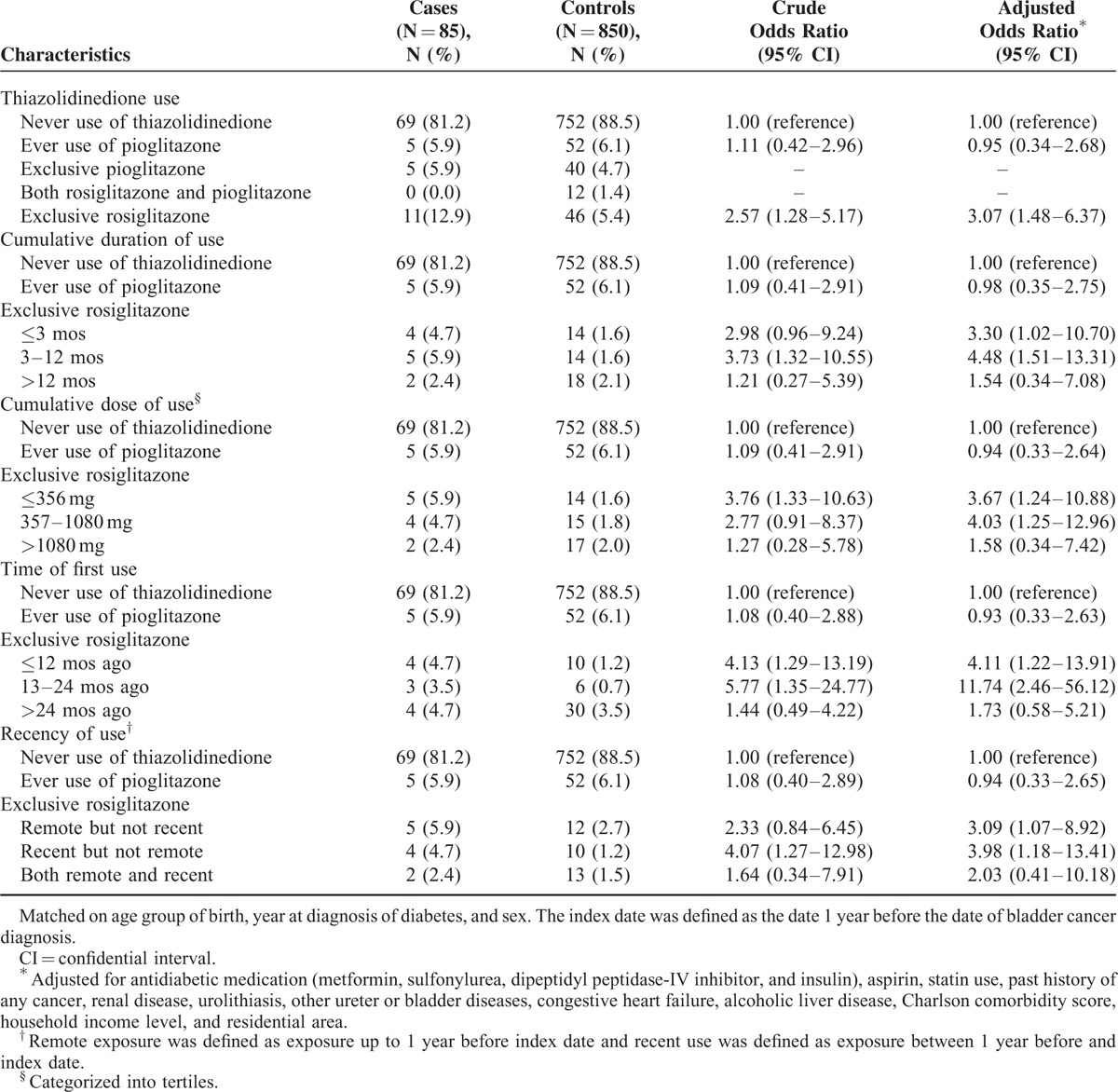
Relationship Between Thiazolidinedione Use and Bladder Cancer

### Rosiglitazone Use was Independently Associated With an Increasing Risk of Bladder Cancer

The association between rosiglitazone use and bladder cancer incidence was evaluated (Supplementary Table 1). Subjects who had taken rosiglitazone at any point had a higher incidence of bladder cancer (AOR 2.36; 95% CI 1.14–4.87) compared with patients who had never been exposed to rosiglitazone. The cumulative duration and doses of rosiglitazone use showed similar patterns to that observed for exclusive rosiglitazone use. Consistent use for rosiglitazone less than 12 months and a cumulative dose less than 2040 mg corresponded to 4 mg/day for 17 months and, were associated with higher risks of bladder cancer. However, a significant increase of bladder cancer incidence was not observed among subjects who had used pioglitazone for any time period (Supplementary Table 2).

### Sensitivity Analysis

The results of the sensitivity analysis did not show any differences from those of the main analysis. First, after excluding 13 case patients via diagnostic procedure or test (biopsy, bladder tumor antigen test, and cystoscopy) and 3 case patients due to early death within 6 months after the diagnosis of bladder cancer, exclusive rosiglitazone use raised the incidence of bladder cancer by approximately threefold compared with no use of any TZD (AOR 3.15, 95% CI 1.34–7.37, Supplementary Table 3 **)**. Second, when we eliminated 17 case patients with any cancer history prior to the bladder cancer diagnosis, exclusive rosiglitazone use raised the incidence of bladder cancer by approximately 3-fold compared with no use of any TZD (AOR 5.08; 95% CI 2.09–12.37, Supplementary Table 4).

### Discussion and Conclusions

Recent studies concluded that pioglitazone use was not associated with a significantly increased risk of bladder cancer.^10,13^ Our group also previously reported no relationship between pioglitazone use and the incidence of bladder cancer in Korean patients with diabetes.^20^ In contrast, research on the impact of rosiglitazone and bladder cancer risk is limited. According to a subsidiary analysis included in a pioglitazone study, rosiglitazone use increased bladder cancer risk in a statistically insignificant way.^7,8^ Furthermore, those studies did not compute the accumulative dose or duration of rosiglitazone use. The current study was conducted to identify the relationship between risk of bladder cancer and TZD use (pioglitazone and rosiglitazone) in T2DM patients. We used a nest-control model f, an incident diabetic cohort and found that rosiglitazone use increased bladder cancer risk. The risk was higher for subjects who had continuously using rosiglitazone for less than 1 year and recent users. However, we found that pioglitazone use was not related to the risk of bladder cancer, which is consistent with the above studies.

Patients with diabetes are likely to have a modestly increased risk of bladder cancer.^6^ In our study of 47,738 T2DM patients, 120 incident bladder cancer patients were identified (215 per 100,000 person). This is a higher incidence than in general populations; the overall rate for bladder cancer in Korea was 4.6 per 100,000 person in 2011.^21^ The distribution of bladder cancer stratified by age peaked at 70 to 79 years old, which is the same as that of the general Korean populations.^21^ It was not known whether there was a difference between bladder cancer characteristics in T2DM and general population. In addition, the mechanism underlying bladder cancer in T2DM is not clearly understood. The incidence of bladder cancer is likely to be increased in a high-risk group such as T2DM.^22,23^ According to a Taiwanese study, older age, male sex, pre-existing nephropathy, and urolithiasis were associated with greater bladder cancer risk.^22^ Renal diseases did not increase the bladder cancer incidence in our study. However, we also found more urolithiasis, a well-established form of bladder tumorigenesis,^24^ significantly increased bladder cancer risk (OR 1.75; 95% CI 1.04–2.97). Furthermore, we set latent period of bladder cancer for 1 year and 3 months to determine whether rosiglitazone acts as an initiator or promotor. Higher incidence of bladder cancer for patients with exposure ≤24 months and recent rosiglitazone use was observed in both settings. These results implied that rosiglitazone might act as a promoter, which is in a line with the findings of an in vivo study.^25^ Given that T2DM patients might be prone to bladder cancer; rosiglitazone use might amplify the process in high-risk patients.

Our finding of a high incidence of bladder cancer in subjects who have used rosiglitazone for less than 1 year is inconsistent with previous studies that demonstrated a dose-response relationship of bladder cancer and rosiglitazone use.^23,26^ Long-term exposure to rosiglitazone from more than 2 to 5 years was associated with a higher risk of bladder cancer. However, the design and settings of previous studies were different from ours. Patients in the United Kingdom with pre-existing T2DM who were newly prescribed rosiglitazone were enrolled in a retrospective cohort study, which is dissimilar to nested case-control study approaches.^23^ In a Taiwanese nested case-control study, the index date for cases was defined as the date of first hospitalization for bladder cancer and included incident T2DM patients treated with rosiglitazone regardless of treatment duration.^26^ No latent period for bladder cancer was considered in either of those studies. As cancer would not appear in such a short period, the setting of the latent period for cancer would be very important and could influence the result. In addition, no dose–response relationship and the risk peak at less than 1 year of exposure stays support the hypothesis that rosiglitazone might act as a promoter and have an early effect of bladder cancer. It is also possible that rosiglitazone could promote bladder cancer in subjects with high sensitivity who develop bladder cancer during the early period of use.

The different effects of pioglitazone and rosiglitazone on bladder cancer are explained by their effects on PPAR gamma. Rosiglitazone is considered a pure PPAR gamma agonist, whereas pioglitazone is both a PPAR alpha and gamma agonist.^27^ Proliferator-activated receptor alpha controls fatty acid catabolism, inflammatory response, and homeostasis in liver.^28–30^ Proliferator-activated receptor gamma regulates adipogenesis, insulin action, and plays roles in cell differentiation, and proliferation.^31,32^ This disparity could explain the different effects on lipid metabolism in previous studies.^27,33^ Moreover, PPAR gamma mediates the up-regulation of vascular endothelial growth factor, which plays a critical role in tumor angiogenesis.^34^ And oxidized low-density lipoprotein stimulates vascular endothelial growth factor expression via PPAR gamma pathway activation.^35^ Taken together, the potency of PPAR gamma agonism exerted by the 2 drugs might result in different effects regarding bladder cancer.

There are several limitations of this study. First, several variables that could be associated with bladder cancer incidence, such as smoking, alcohol consumption, obesity, and exposure to environment and occupational chemicals are not measured in NHIS-NSC survey. For smoking, a well-known risk factor for bladder cancer,^36^ we assumed that chronic lower respiratory disease indirectly represented smoking cigarettes and adjusted this in the final risk for bladder cancer. Likewise, no laboratory tests (glycated hemoglobin, fasting serum glucose, postprandial glucose) of diabetic parameters were available, so we could not assess whether glycemic fluctuation or diabetes severity influenced bladder cancer risk. Thiazolidinedione is mostly prescribed as the second medication for patients whose T2DM is poorly controlled with metformin, so the population of current study would not be in the early disease stage. We matched cases and controls for age, sex, and date of T2DM diagnosis; therefore, the effect of those variables would be minimized. Second, as the data source was derived from a claim dataset, the actual adherence rates of medication are not reflected. This weak point was already noticed in a similar previous design.^26^ In this study, we assumed all medications prescribed were taken by the study patients and excluded those who failed to fill 2 or more prescriptions within 6 months. Third, the number of case patients was relatively small. We made an effort to improve on the retrospective cohort study by employing a nested case-control model. In addition, the study population was restricted to newly diagnosed T2DM patient; this allowed us to assess the pure effect of TZDs and let the case patients have a 1-year latent period of bladder cancer development. Because of these strict criteria, the final number of cases was smaller. Despite this, the results were statistically significant.

There are major strengths of the current study. First, the nationwide database included a large population of incident T2DM patients and contained both outpatient and inpatient references, which were not included in a previous study.^7^ Second, the concept of the current study model is an aggregate of preceding analyses, so this nested case-control study is more sophisticated and provides higher accuracy. As described above, we determined the latent window period for bladder cancer after the diagnosis of T2DM and applied strict inclusion and exclusion criteria.

In conclusion, the present study shows that rosiglitazone but not pioglitazone increased bladder cancer risk in T2DM patients. In addition, bladder cancer incidence in rosiglitazone users was higher among those who had been taking the drug for less than 1 year. This suggests that rosiglitazone might promote the development of bladder cancer, but long-term studies are needed to assess the clear causality and mechanisms regarding rosiglitazone use and bladder cancer risk. Furthermore, it would be interesting to determine differences of bladder cancer in the general population and rosiglitazone users. If there is a clear relationship between rosiglitazone and bladder cancer, a rosiglitazone substitute might be prescribed for high-risk T2DM patients.

## Supplementary Material

Supplemental Digital Content
