# Supplementary material for: Rosiglitazone Use and the Risk of Bladder Cancer in Patients With Type 2 Diabetes
Source: Medicine (Baltimore). 2016 Feb 12;95(6):e2786. doi: 10.1097/MD.0000000000002786 (PMC4753931; doi:10.1097/MD.0000000000002786)
Supplement: Supplemental Digital Content [file medi-95-e2786-s001.pdf]

Supplementary Table 1. Relationship between rosiglitazone use and bladder cancer

Characteristics	Cases (N=85), N(%)	Controls (N=850), N(%)	Crude odds ratio (95% CI)	Adjusted odds ratio* (95% CI)
Rosiglitazone use				
Never use	74 (87.1)	792 (93.2)	1.00 (reference)	1.00 (reference)
Ever use	11 (12.9)	58 (6.8)	2.03 (1.02-4.02)	2.36 (1.14-4.87)
Cumulative duration of use				
Never use of rosiglitazone	74 (87.1)	792 (93.2)	1.00 (reference)	1.00 (reference)
Ever use of rosiglitazone			1.09 (0.41-2.91)	0.98 (0.35-2.75)
≤3 months	4 (4.7)	15 (1.8)	2.74 (0.90-8.39)	3.18 (0.99-10.20)
3-12 months	5 (5.9)	16 (1.9)	3.28 (1.17-9.19)	3.84 (1.31-11.24)
>12 months	2 (2.4)	27 (3.2)	0.79 (0.18-3.41)	0.86 (0.18-4.07)
Cumulative dose of use §				
Never use of rosiglitazone	74 (87.1)	792 (93.2)	1.00 (reference)	1.00 (reference)
Ever use of rosiglitazone				
≤408 mg	5 (5.9)	18 (2.1)	2.93 (1.07-8.08)	3.07 (1.06-8.87)
409-2040 mg	5 (5.9)	18 (2.1)	2.89 (1.06-7.87)	4.36 (1.49-12.71)
>2040 mg	1 (1.2)	22 (2.6)	0.48 (0.06-3.65)	0.41 (0.05-3.61)
Time of first use				
Never use of rosiglitazone	74 (87.1)	792 (93.2)	1.00 (reference)	1.00 (reference)
Ever use of rosiglitazone				
≤12 months ago	4 (4.7)	10 (1.2)	4.10 (1.28-13.09)	4.06 (1.20-13.70)

13-24 months ago	3 (3.5)	6 (0.7)	5.57 (1.31-23.73)	11.08 (2.35-52.29)
>24 months ago	4 (4.7)	42 (4.9)	0.99 (0.34-2.88)	1.08 (0.34-3.39)
Recency of use†				
Never use of rosiglitazone	74 (87.1)	792 (93.2)	1.00 (reference)	1.00 (reference)
Ever use of rosiglitazone				
Remote but not recent	5 (5.9)	35 (4.1)	1.50 (0.55-4.04)	1.83 (0.62-5.38)
Recent but not remote	4 (4.7)	10 (1.2)	4.03 (1.27-12.86)	3.90 (1.16-13.11)
Both remote and recent	2 (2.4)	13 (1.5)	1.63 (0.34-7.82)	1.98 (0.39-9.94)

Matched on age group of birth, year at diagnosis of diabetes, and sex.

The index date was defined as the date one year before the date of bladder cancer diagnosis.

*Adjusted for antidiabetic medication (metformin, sulfonylurea, dipeptidyl peptidase-IV inhibitor, insulin), aspirin, statin use, past history of any cancer, renal disease, urolithiasis, other ureter or bladder diseases, congestive heart failure, alcoholic liver disease, Charlson comorbidity score, household income level and residential area.

§Categorized into tertiles.

†Remote exposure was defined as exposure up to one year before index date and recent use was defined as exposure between one year before and index date.

Abbreviations: CI: confidential interval.

Supplementary Table2. Relationship between pioglitazone use and bladder cancer

Characteristics	Cases (N=85), N(%)	Controls (N=850),N(%)	Crude odds ratio (95% CI)	Adjusted odds ratio* (95% CI)

Pioglitazone use				
Never use	80 (94.1)	798 (93.9)	1.00 (reference)	1.00 (reference)
Ever use	5 (5.9)	52 (6.1)	0.96 (0.36-2.53)	0.75 (0.26-2.15)
Cumulative duration of use				
Never use of pioglitazone	80 (94.1)	798 (93.9)	1.00 (reference)	1.00 (reference)
Ever use of pioglitazone				
≤3 months	3 (3.5)	13 (1.5)	2.23 (0.64-7.83)	1.52 (0.37-6.26)
3-12 months	1 (1.2)	13 (1.5)	0.75 (0.99-6.07)	0.88 (0.10-7.75)
>12 months	1 (1.2)	26 (3.1)	0.37 (0.05-2.86)	0.31 (0.04-2.51)
Cumulative dose of use				
Never use of pioglitazone	80 (94.1)	798 (93.9)	1.00 (reference)	1.00 (reference)
Ever use of pioglitazone				
≤4125 mg	4 (4.7)	25 (2.9)	1.58 (0.53-4.68)	1.38 (0.41-4.59)
>4125 mg	1 (1.2)	27 (3.2)	0.36 (0.05-2.75)	0.26 (0.03-2.12)
Time of first use				
Never use of pioglitazone	80 (94.1)	798 (93.9)	1.00 (reference)	1.00 (reference)
Ever use of pioglitazone				
≤12 months ago	2 (2.4)	12 (1.4)	1.63 (0.36-7.29)	1.60 (0.34-7.47)
13-24 months ago	1 (1.2)	10 (1.2)	0.99 (0.13-7.76)	1.38 (0.17-11.16)
>24 months ago	2 (2.4)	30 (3.5)	0.66 (0.15-2.89)	0.36 (0.07-1.85)
Recency of use†				
Never use of pioglitazone	80 (94.1)	798 (93.9)	1.00 (reference)	1.00 (reference)

Ever use of pioglitazone				
Remote but not recent	2 (2.4)	24 (2.8)	0.83 (0.19-3.64)	0.47 (0.09-2.60)
Recent but not remote	2 (2.4)	12 (1.4)	1.63 (0.37-7.31)	1.58 (0.34-7.41)
Both remote and recent	1 (1.2)	16 (1.9)	0.62 (0.08-4.81)	0.59 (0.07-4.86)

Matched on age group of birth, year at diagnosis of diabetes, and sex.

The index date was defined as the date one year before the date of bladder cancer diagnosis.

*Adjusted for antidiabetic medication (metformin, sulfonylurea, dipeptidyl peptidase-IV inhibitor, insulin), aspirin, statin use, past history of any cancer, renal disease, urolithiasis, other ureter or bladder diseases, congestive heart failure, alcoholic liver disease, Charlson comorbidity score, household income level and residential area.

§Categorized into tertiles.

†Remote exposure was defined as exposure up to one year before index date and recent use was defined as exposure between one year before and index date.

Abbreviations: CI: confidential interval.

Supplementary Table 3. Relationship of exclusive rosiglitazone use to bladder cancer : excluding 16 case patients selected by diagnostic test criteria and early death criteria

	Cases (N=69), N (%)	Controls (N=690), N (%)	Crude odds ratio (95% CI)	Adjusted odds ratio* (95% CI)
Never use of thiazolidinediones	55 (79.7)	597 (86.5)	1.00 (reference)	1.00 (reference)

Ever use of pioglitazone	5 (7.2)	58 (8.4)	0.97 (0.37–2.55)	0.82 (0.30–2.24)
Exclusive pioglitazone	5 (7.2)	50 (7.2)	–	–
Both rosiglitazone and pioglitazone	0 (0.0)	8 (1.2)	–	–
Exclusive rosiglitazone	9 (13.0)	35 (5.1)	2.84 (1.28–6.32)	3.15 (1.34–7.37)

Matched on age group of birth, year at diagnosis of diabetes, and sex

The index date was defined as the date one year before the date of bladder cancer diagnosis

*Adjusted for diabetes medication (metformin, sulfonylurea, dipeptidyl peptidase-IV inhibitor, insulin), aspirin, statin use, past history of any cancer, renal disease, urolithiasis, other ureter or bladder diseases, congestive heart failure, alcoholic liver disease, Charlson comorbidity score, household income level and residential area

Abbreviations: CI: confidential interval.

Supplementary Table 4. Relationship of exclusive rosiglitazone use to bladder cancer : restricted to subjects without any cancer prior to the bladder cancer diagnosis

	Cases (N=68), N (%)	Controls (N=680), N (%)	Crude odds ratio (95% CI)	Adjusted odds ratio* (95% CI)
Never use of thiazolidinediones	54 (79.4)	601 (88.4)	1.00 (reference)	1.00 (reference)
Ever use of pioglitazone	5 (7.4)	55 (8.1)	1.05 (0.40–2.72)	0.97 (0.36–2.61)
Exclusive pioglitazone	5 (7.4)	44 (6.5)	–	–
Both rosiglitazone and pioglitazone	0 (0.0)	11 (1.6)	–	–
Exclusive rosiglitazone	9 (13.2)	24 (3.5)	4.07 (1.81–9.14)	5.08 (2.09–12.37)

Matched on age group of birth, year at diagnosis of diabetes, and sex

The index date was defined as the date one year before the date of bladder cancer diagnosis

*Adjusted for diabetes medication (metformin, sulfonylurea, dipeptidyl peptidase-IV inhibitor, insulin), aspirin, statin use, past history of any cancer, renal disease, urolithiasis, other ureter or bladder diseases, congestive heart failure, alcoholic liver disease, Charlson comorbidity score, household income level and residential area

Abbreviations: CI: confidential interval.
